# Reclassification of the Specialized Metabolite Producer Pseudomonas mesoacidophila ATCC 31433 as a Member of the Burkholderia cepacia Complex

**DOI:** 10.1128/JB.00125-17

**Published:** 2017-06-13

**Authors:** E. Joel Loveridge, Cerith Jones, Matthew J. Bull, Suzy C. Moody, Małgorzata W. Kahl, Zainab Khan, Louis Neilson, Marina Tomeva, Sarah E. Adams, Andrew C. Wood, Daniel Rodriguez-Martin, Ingrid Pinel, Julian Parkhill, Eshwar Mahenthiralingam, John Crosby

**Affiliations:** a Department of Chemistry, Swansea University, Swansea, United Kingdom; b School of Chemistry, Cardiff University, Cardiff, United Kingdom; c School of Biosciences, Cardiff University, Cardiff, United Kingdom; d Department of Biosciences, Swansea University, Swansea, United Kingdom; e School of Medicine, Swansea University, Swansea, United Kingdom; f Wellcome Trust Sanger Institute, Wellcome Trust Genome Campus, Hinxton, United Kingdom; g School of Chemistry, University of Bristol, Bristol, United Kingdom; University of Tennessee at Knoxville

**Keywords:** genome, identification, antibiotic resistance, metal resistance, antibacterial, biosynthesis, bulgecin

## Abstract

Pseudomonas mesoacidophila ATCC 31433 is a Gram-negative bacterium, first isolated from Japanese soil samples, that produces the monobactam isosulfazecin and the β-lactam-potentiating bulgecins. To characterize the biosynthetic potential of P. mesoacidophila ATCC 31433, its complete genome was determined using single-molecule real-time DNA sequence analysis. The 7.8-Mb genome comprised four replicons, three chromosomal (each encoding rRNA) and one plasmid. Phylogenetic analysis demonstrated that P. mesoacidophila ATCC 31433 was misclassified at the time of its deposition and is a member of the Burkholderia cepacia complex, most closely related to Burkholderia ubonensis. The sequenced genome shows considerable additional biosynthetic potential; known gene clusters for malleilactone, ornibactin, isosulfazecin, alkylhydroxyquinoline, and pyrrolnitrin biosynthesis and several uncharacterized biosynthetic gene clusters for polyketides, nonribosomal peptides, and other metabolites were identified. Furthermore, P. mesoacidophila ATCC 31433 harbors many genes associated with environmental resilience and antibiotic resistance and was resistant to a range of antibiotics and metal ions. In summary, this bioactive strain should be designated B. cepacia complex strain ATCC 31433, pending further detailed taxonomic characterization.

**IMPORTANCE** This work reports the complete genome sequence of Pseudomonas mesoacidophila ATCC 31433, a known producer of bioactive compounds. Large numbers of both known and novel biosynthetic gene clusters were identified, indicating that P. mesoacidophila ATCC 31433 is an untapped resource for discovery of novel bioactive compounds. Phylogenetic analysis demonstrated that P. mesoacidophila ATCC 31433 is in fact a member of the Burkholderia cepacia complex, most closely related to the species Burkholderia ubonensis. Further investigation of the classification and biosynthetic potential of P. mesoacidophila ATCC 31433 is warranted.

## INTRODUCTION

Pseudomonas mesoacidophila was isolated from Japanese soil samples in the late 1970s (1). It is known to produce the sulfamate monobactam isosulfazecin (1) and the bulgecins (2), a group of sulfated glycopeptides that inhibit lytic transglycosylases (3) and metallo-β-lactamases (4) and potentiate β-lactam activity (2, 5). Pseudomonas and other soil Gram-negative bacterial genera, such as Stenotrophomonas and Burkholderia, often show high levels of resistance to antibiotics (6). For example, Pseudomonas aeruginosa is a common nosocomial pathogen with reduced susceptibility to a range of antibiotics, due to low membrane permeability, chromosomal multidrug efflux pumps, overexpressed β-lactamases, and the acquisition of multidrug resistance plasmids (6, 7). Stenotrophomonas maltophilia is an emerging nosocomial pathogen (8), and several Burkholderia species, even environmental isolates, are resistant to a wide range of antibiotic classes (9). Species of both Pseudomonas and Burkholderia produce an array of specialized bioactive metabolites (10, 11). Despite these findings, the full biosynthetic potential and antibiotic susceptibility of P. mesoacidophila have not been reported. Here, we report the complete genome sequence of P. mesoacidophila, and we show that this species shares an antimicrobial-resistant phenotype characteristic of Burkholderia and should be reclassified phylogenetically as a member of the Burkholderia cepacia complex.

## RESULTS

### Genome sequencing and taxonomic placement of P. mesoacidophila ATCC 31433 within the Burkholderia genus.

**FIG 1:**
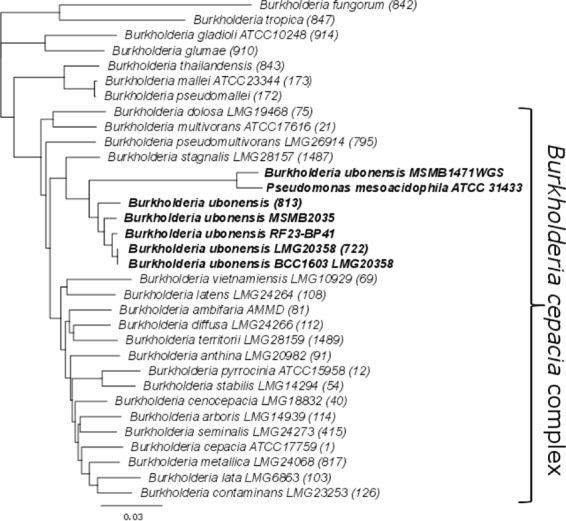
Phylogeny of P. mesoacidophila ATCC 31433 within the genus Burkholderia. The analysis was based on the sequences of six MLST alleles (*trpB*, *recA*, *lepA*, *gyrB*, *gltB*, and *atpD*). The PubMLST isolate identification numbers are shown in parentheses after the species names.

Previous studies showed P. mesoacidophila ATCC 31433 to be a producer of specialized bioactive metabolites (1, 2, 12). There is only one known example of this species, with no nucleotide sequences reported. Amplification and sequencing of 16S, *recA*, and *gyrB* genes indicated that these sequences were closely related to those of Burkholderia species (data not shown) (13). To characterize this isolate, we obtained a complete genome sequence using PacBio technology. The sequence assembled into four contigs, with a total size of 7.84 MB. The sequences of six multilocus sequence typing (MLST) loci from the genome were compared to those of characterized Burkholderia species. P. mesoacidophila ATCC 31433 clustered most closely with Burkholderia ubonensis within the Burkholderia cepacia complex (Fig. 1), which confirms its misclassification.

**FIG 2:**
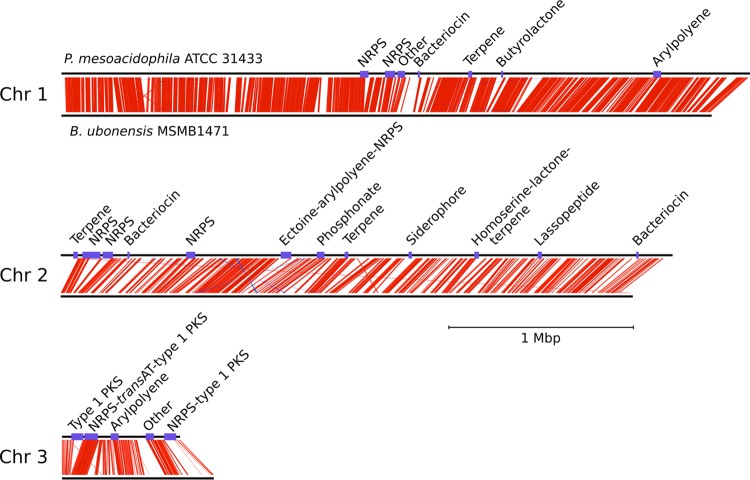
Genome sequence comparison of P. mesoacidophila ATCC 31433 and B. ubonensis MSMB1471. Each chromosome (Chr) is displayed separately and to scale (as indicated), with P. mesoacidophila ATCC 31433 above and B. ubonensis MSMB1471 below. Regions of homologous sequences are linked with red lines. AntiSMASH-predicted gene clusters are shown in purple. The smallest contig of the P. mesoacidophila ATCC 31433 genome is not shown, because it neither had homology with B. ubonensis MSMB1471 nor contained any antiSMASH-predicted biosynthetic pathways.

The genome sequence of P. mesoacidophila ATCC 31433 was compared to that of B. ubonensis MSMB1471 (GenBank accession numbers NZ_CP013462, NZ_CP013463, NZ_CP013464, and NZ_CP013465) (Fig. 2). Both genomes were organized similarly in three chromosomes, each encoding rRNA, and were largely syntenic. The B. ubonensis MSMB1471 and P. mesoacidophila ATCC 31433 genomes shared an average nucleotide identity (ANI) of 94.7%, which falls below the proposed species-level cutoff value of 95% (14). These data suggest that P. mesoacidophila ATCC 31433, while clearly a member of the B. cepacia complex and related to B. ubonensis, may yet be a novel species within this taxonomic subgroup of Burkholderia.

### General features of the P. mesoacidophila ATCC 31433 genome.

Annotation of the genome revealed numerous elements that suggest resilience to environmental conditions. Genes are present encoding detoxification proteins, such as nitric oxide dioxygenase (flavohemoglobin), superoxide dismutase, catalase, catalase/peroxidase, thiol peroxidase, alkylhydroperoxidase, alkylhydroperoxide reductase, alkylsulfonate monooxygenase, and transport proteins, and a range of proteins involved in sulfite metabolism, DNA repair, and the osmotic stress response. Genes and gene clusters required for uptake and metabolism of chitin, collagen, pectin, phenylpropanoids, phospholipids, glycerol, arabinose, ribose, fucose, xylose, trehalose, polyols, glycolate, lactate, 2-ketogluconate, hydantoin, allantoin, heme, taurine, putrescine, and methylamine were all identified. Siderophore synthetases, transport proteins, and receptors are present, along with several other proteins involved in metal homeostasis. Genes encoding a wide range of antibiotic resistance proteins and efflux pumps are also present.

Interactions with the environment are also mediated by the chaperone-usher fimbriae, Flp pili, type IV pili, adhesins, and biofilm-forming proteins (PgaABCD and PelABCDEFG) identified, while motility is suggested by genes encoding flagellar components. Lipopolysaccharide biosynthesis genes include those for O-antigen biosynthesis and lipid A modification. A number of genes and gene clusters involved in specialized metabolite biosynthesis were indicated. In addition, the genome contains several phage proteins, transposases, and Tra and Trb conjugative transfer proteins.

P. mesoacidophila harbors genes encoding secreted enzymes and toxins involved in virulence and interactions with hosts, such as phospholipases (including a phosphatidylinositol-specific phospholipase C seen in other Burkholderia species [15]), secreted collagenase, LasA protease, secreted zinc metalloprotease, pectin-degrading polygalacturonase, HigAB, RelBE, YgiTU, and VapBC antitoxin-toxin pairs, RTX toxin, Phd antitoxin, and toxin secretion proteins. Components of possible type I, II, III, IV, and VI secretion systems have also been identified.

### Specialized metabolite production by P. mesoacidophila ATCC 31433.

To uncover the secondary metabolite potential of P. mesoacidophila, we analyzed the genome using the antiSMASH tool (16) and performed comparative investigations with other B. ubonensis genomes. This identified 91 putative biosynthetic gene clusters from five isolates. P. mesoacidophila yielded 24 clusters, suggesting 40% more biosynthetic potential than the other B. ubonensis isolates analyzed (Table 1).

**TABLE 1:** Biosynthetic gene clusters from P. mesoacidophila and B. ubonensis

Strain	No. of predicted clusters^*a*^													
	NRPS	PKS containing^*b*^	Terpene	Bacteriocin	Bacteriocin-proteusin	Arylpolyene	Siderophore	Lassopeptide	Homoserine lactone-terpene	Phosphonate	Butyrolactone	Ectoine-arylpolyene-NRPS	Other	Total
B. ubonensis RF23-BP41	3	2	3	1	1	3	0	0	1	1	1	0	1	17
B. ubonensis SMB1471WGS	2	3	3	1	0	4	0	0	1	1	0	0	2	18
B. ubonensis MSMB2035	3	1	3	1	0	2	0	0	1	1	1	0	1	14
B. ubonensis BCC 1603	4	2	3	1	0	3	0	0	1	1	1	0	2	18
P. mesoacidophila ATCC 31433	5	3	3	3	0	2	1	1	1	1	1	1	2	24
Total														91

a The numbers of secondary metabolite clusters in P. mesoacidophila and four closely related isolates of B. ubonensis, as predicted by antiSMASH, are shown.

b PKS-containing clusters include T1PKS, *trans*-AT PKS, other KS, and any mixed cluster containing one of these types.

A small proportion of the biosynthetic clusters identified are required for the biosynthesis of known compounds. Of the nine polyketide synthase (PKS) and nonribosomal peptide synthetase (NRPS) clusters, three are known, three have equivalents in other Burkholderia species, and three have no established equivalents indicated by antiSMASH. The complete PKS cluster for malleilactone production (17) and the NRPS cluster for ornibactin biosynthesis (18) were found, and the NRPS part of the ectoine-arylpolyene-NRPS cluster is similar to the sulfazecin biosynthesis cluster in Pseudomonas acidophila (19), with the exception that the SulM equivalent in P. mesoacidophila has no epimerization domain. Therefore, this group of genes is likely to be responsible for isosulfazecin biosynthesis. The complete four-gene cluster required for biosynthesis of the antifungal pyrrolnitrin (20) is present, although we could not detect production of this metabolite in either culture broth or partially purified cell extracts with liquid chromatography-mass spectrometry (LC-MS). Genes necessary for the synthesis of alkylhydroxyquinolines, which are involved in quorum sensing (21), were found in one of the NRPS clusters.

One of the three terpene clusters constitutes part of the hopanoid biosynthetic pathway described for Burkholderia cenocepacia J2315 (encoding seven proteins with >75% identity to their counterparts HpnCEFGMN and VacJ, plus two proteins not found in that species) (15, 22). A response regulator gene just downstream of the *hpnCEFGMN* cluster encodes a protein 84% identical to Bp1026b_II2523 from Burkholderia pseudomallei, which is involved in thermoregulation and biofilm formation (23). Clusters equivalent (>75% identical at the protein level) to *hpnABH*/*ispH* and *hpnIJKL*/*ompA* in B. cenocepacia J2315 (22) were also found, making up the entire hopanoid biosynthetic pathway. The other two terpene clusters and the homoserine lactone-terpene cluster contain one isolated squalene-hopene cyclase gene and two isolated squalene synthase genes. The homoserine lactone synthase and its associated regulator found in the homoserine lactone-terpene cluster, which are involved in quorum sensing, encode proteins >85% identical to their equivalents in B. cenocepacia J2315 (15, 24).

In addition to core genes encoding fatty acid synthase components >80% identical to those seen in other Burkholderia species (25), two genes encoding cyclopropane fatty acyl phospholipid synthases (known components of Burkholderia cell membranes [26]) and one encoding diffusible factor synthase (90% identical at the protein level to BCAM0581 from B. cenocepacia J2315), which is responsible for production of a fatty acid involved in quorum sensing (27), were found. P. mesoacidophila also produces poly(β-hydroxybutanoate) (1), and poly(β-hydroxyalkanoate) synthase and depolymerase genes were identified.

### Production of bulgecin by P. mesoacidophila.

**FIG 3:**
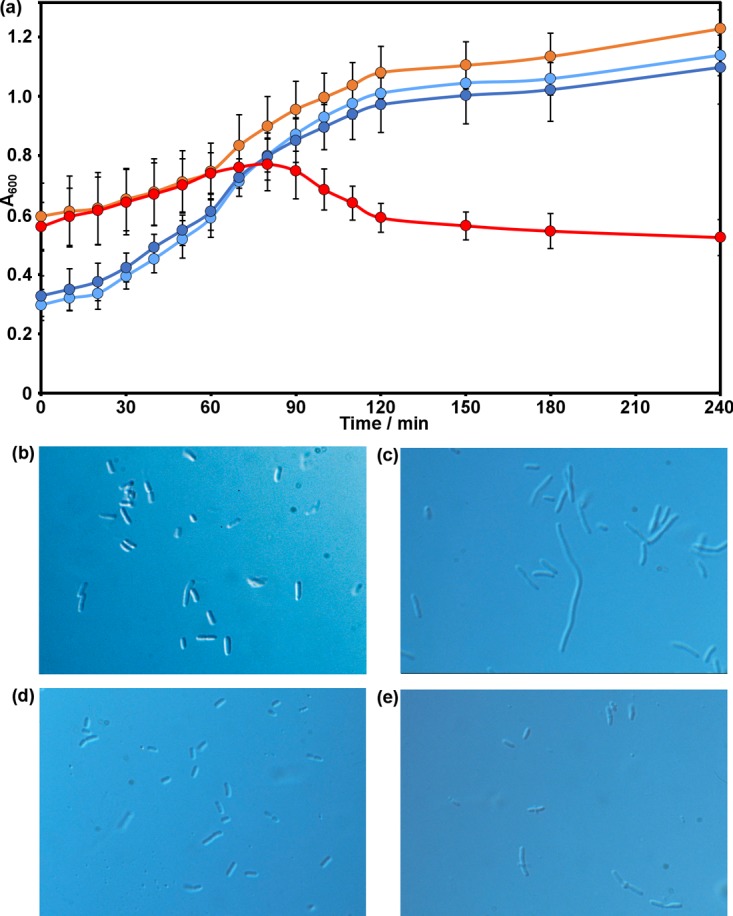
Cefuroxime potentiation and bulge-forming activity. (a) Growth curves of E. coli JM109 in PS medium without (light blue) and with (dark blue) 5 μg ml^−1^ cefuroxime and in P. mesoacidophila culture filtrate without (orange) and with (red) 5 μg ml^−1^ cefuroxime. (b to e) Light microscopy of E. coli JM109 alone (b) and exposed to 5 μg ml^−1^ cefuroxime (c), P. mesoacidophila culture filtrate (d), and a combination of 5 μg ml^−1^ cefuroxime and P. mesoacidophila culture filtrate (e). Cefuroxime potentiation and bulge formation are consistent with the presence of bulgecin (2, 28).

We sought to confirm the presence of bulgecin in the culture broth of P. mesoacidophila ATCC 31433. Bulgecin was shown previously to potentiate the activity of β-lactam antibiotics and to cause bulge formation in Escherichia coli (2, 28). After 78 h of growth at 28°C in PF medium, P. mesoacidophila gave 36 g of cell pellet per liter of culture and reduced the pH of the medium from 7.0 to 5.5 ± 0.5. The medium acquired a distinctive fruity smell and remained translucent even after repeated centrifugation at 6,000 × *g* to remove cells. The culture filtrate of P. mesoacidophila potentiated the lytic activity of cefuroxime against E. coli and caused bulge formation (Fig. 3), as expected if bulgecin were present (2, 28). E. coli JM109 cells exposed to a sublethal concentration of cefuroxime grew as long cell chains, consistent with this compound's selective inhibition of PBP3 and prevention of proper septation (29), whereas cells exposed to the same concentration of cefuroxime in P. mesoacidophila culture broth developed bulges at the sites of septation (2, 28).

### Antibiotic and metal resistance of P. mesoacidophila.

P. mesoacidophila was resistant to a wide range of antibiotics (Table 2), including the antipseudomonal aminoglycoside tobramycin, the carbapenems, the monobactam aztreonam, and polymyxin B. Of the 24 antibiotics tested, only the antipseudomonal β-lactams piperacillin and ceftazidime showed clear activity against P. mesoacidophila, with the fluoroquinolone ciprofloxacin and the fourth-generation cephalosporin cefepime showing intermediate activity. In a few cases, the zone diameters in disc diffusion assays were close to the breakpoint value (with some replicates indicating resistance and others suggesting a degree of susceptibility), while the broth microdilution method clearly indicated resistance. Burkholderia species are known to give poor correlation between the two methods (30). E. coli JM109, which was tested simultaneously, was susceptible to all antibiotics assayed except erythromycin and nalidixic acid, as expected (data not shown).

**TABLE 2:** Antibiotic susceptibility of P. mesoacidophila ATCC 31433, determined by the broth microdilution and disc diffusion methods

Antibiotic	Microdilution method			Disc diffusion method				Resistance
	MIC^*a*^ (μg ml^−1^)	Breakpoint^*b*^		Dose (μg)	Zone of inhibition (mm)	Breakpoint^*b*^		
		R (>)	S (≤)			R (≤)	S (≥)	
Amoxicillin	>128	(8)	(8)	30	0	(14)	(15)	R
Co-amoxiclav	>128	(8)	(8)	20/10	0	(20)	(21)	R
Ampicillin	ND			25	0	(14)	(15)	R
Carbenicillin	>128			100	12	12	13	R
Piperacillin	8	16	16	75	37	24	25	S
Cefuroxime	>128	(8)	(8)	30	21	(19)	(20)	R
Cefoxitin	>128	(8)	(8)	30	8	(22)	(23)	R
Cefotaxime	16	(2)	(1)	30	23	26	27	R
Ceftazidime	8	8	8	30	29	23	24	S
Cefepime	4	(4)	(1)	30	31	(26)	(30)	I
Imipenem	ND	8	4	10	12	16	23	R
Meropenem	16	8	2	10	19	15	20	R
Aztreonam	128	16	1	30	18	19	36	R
Erythromycin	>128			30	0			R
Tetracycline	64			10	0	(23)	(24)	R
Chloramphenicol	32	(8)	(8)	30	17	(20)	(21)	R
Streptomycin	ND			10	0			R
Kanamycin	64			30	18			R
Gentamicin	128	4	4	10	9	17	18	R
Tobramycin	ND	4	4	10	11	19	20	R
Nalidixic acid	32	(16)	(16)	30	20	(17)	(18)	R
Ciprofloxacin	0.5	1	0.5	5	26	19	30	I
Rifampin	16			30	18			
Polymyxin B	64	4	4	25	14			R

a ND, not determined.

b Breakpoints are for Pseudomonas spp.; breakpoints in parentheses are for Enterobacteriaceae. The polymyxin B breakpoint is for colistin (polymyxin E). R, resistant; I, intermediate; S, susceptible.

P. mesoacidophila has predicted genes for β-lactamases from classes A and C, along with other proteins containing β-lactamase domains. Inducible β-lactamase activity was demonstrated using cefoxitin (Table 3), whereas no inducible β-lactamase activity and much greater susceptibility to cefoxitin were seen for E. coli JM109. Two of the genes thought to encode class A β-lactamases have 63% and 54% identity with the genes encoding the known inducible class A β-lactamase from Burkholderia mallei and B. pseudomallei (31).

**TABLE 3:** Induction of β-lactamase activity in P. mesoacidophila ATCC 31433 by cefoxitin

Cefoxitin concentration (μg ml^−1^)^*a*^	Nitrocefin hydrolysis rate (nM s^−1^)	Fold induction
0	22.7 ± 3.4	1
1	28.7 ± 1.4	1.3 ± 0.2
10	68.5 ± 1.2	3.0 ± 0.5
100	300.8 ± 29.9	13.2 ± 2.4

a The growth of P. mesoacidophila ATCC 31433 was unaffected by cefoxitin at the concentrations used.

Resistance to polymyxin B is rare among Gram-negative species, including pseudomonads, but is well known among Burkholderia species, in which the modification of lipid A prevents antibiotic binding (32). Indeed, the Arn gene cluster, which is responsible for the biosynthesis of 4-amino-4-deoxyarabinose and its incorporation into lipid A (33, 34), has been identified. This outer membrane modification also confers decreased susceptibility to aminoglycosides, which, together with a predicted aminoglycoside phosphotransferase (also seen in B. cenocepacia strain J2315 [15]) and several membrane efflux pumps, accounts for the marked resistance to this class of antibiotics (35, 36).

The genome also carries genes encoding CreBCD colicin resistance proteins, a bleomycin (glycopeptide) resistance protein (63% identical to the Tn*5*-mediated bleomycin resistance protein [37], including conservation of the bleomycin-binding regions), phosphinothricin *N*-acetyltransferase (36% identical to phosphinothricin *N*-acetyltransferase from Brucella ovis [GenBank accession number WP_006155257; PDB accession number 5DWN], with significant conservation of the active site), various acyltransferases and glycosyltransferases that may play roles in resistance, and an undecaprenyl diphosphatase associated with bacitracin resistance (38). Genes potentially encoding putative trimethoprim-resistant dihydrofolate reductase FolM (25% identity with FolM from E. coli) and chloramphenicol phosphotransferase (27% identical to the N-terminal two-thirds of chloramphenicol phosphotransferase from Streptomyces venezuelae [GenBank accession number CCA57350 {39}; PDB accession number 5DWN], with the active site substantially conserved) were also found.

Genes encoding efflux pumps from the major facilitator superfamily (MFS); the ATP-binding cassette (ABC) family, including a putative MacAB (macrolide resistance) system; the resistance-nodulation-division (RND) family, including putative AcrAB (acriflavin resistance), CmeABC, and NodT family pumps; the drug/metabolite transporter (DMT) family, including putative EmrE (ethidium bromide/methyl viologen resistance) proteins; the LysE family; and the multiantimicrobial extrusion (MATE) family were identified. Efflux pumps specifically annotated as bicyclomycin (peptide) resistance and fosmidomycin (phosphonate) resistance proteins are present.

P. mesoacidophila was also resistant to metal ions (Table 4), with the order of resistance for the transition metals tested being as follows: Mn^2+^ > Ni^2+^ > Zn^2+^ > Fe^3+^ ≈ Cu^2+^ > Co^2+^ ≫ Hg^2+^. E. coli JM109, in comparison, was sensitive to all transition metals tested, with MICs for MnCl_2_ and HgCl_2_ of <4 μM. Several predicted genes encoding heavy metal resistance proteins were found; these included arsenate reductase and other arsenic resistance proteins, the chromate transporter ChrAB, the cobalt/zinc/cadmium transporter CzcABCD (with a two-component regulator similar to that mediating metal resistance in B. pseudomallei [40]), the copper resistance proteins CopC and CopD, the HoxN/HupN/NixA family nickel/cobalt transporter, a lead/cadmium/zinc/mercury transporter, the manganese transport protein MntH, the magnesium/cobalt efflux pump CorAC, and the tellurium resistance proteins TerA, TerB, TerC, and TerD.

**TABLE 4:** Metal ion susceptibility of P. mesoacidophila ATCC 31433, as determined by the broth microdilution method

Salt^*a*^	MIC (mg ml^−1^)	MIC (mM)
NaCl	>15	>257
KCl	>10	>133
MgCl_2_	>10	>105
CaCl_2_	>10	>90
MnCl_2_·4H_2_O	5	25
FeCl_3_	1	6
CoCl_2_·6H_2_O	0.25	1
NiCl_2_	1.25	10
CuCl_2_·2H_2_O	1	6
ZnCl_2_	1	7
HgCl_2_	0.008	0.03

a All broths contained 5 mg ml^−1^ NaCl.

## DISCUSSION

The 7.8-MB genome of strain ATCC 31433 was sequenced and found to comprise three chromosomal replicons and one plasmid. MLST analysis revealed that strain ATCC 31433, rather than being a pseudomonad, is a member of the Burkholderia cepacia complex and is closely related to B. ubonensis, although it may still be a distinct species. Further analysis of the bioactivity, taxonomy, and genomics of strain ATCC 31433 is warranted under its reclassification as a member of the B. cepacia complex.

Strain ATCC 31433 harbors many genes associated with environmental resilience and antibiotic resistance, including a wide range of efflux pumps. Antibiotic and metal susceptibility testing confirmed the resistant phenotype. Only 2 of the 24 antibiotics tested (piperacillin and ceftazidime) showed clear activity against strain ATCC 31433, and similar resistance to transition metal ions was observed. While many of the predicted heavy metal resistance genes encode efflux pumps, other detoxification systems were also seen. Several environmental Burkholderia strains have been described as metal resistant, although most of those strains are actually less tolerant of metal ions than P. mesoacidophila (40–43). The antibiotic resistance found in strain ATCC 31433 is similar to that seen in Burkholderia species. Indeed, several Burkholderia species with high-level β-lactam resistance have been identified (9, 44). Pseudomonads are generally susceptible to monobactams, carbapenems, cefepime, aminoglycosides, and polymyxin B, whereas strain ATCC 31433 is resistant to all of these compounds, including the antipseudomonal aminoglycoside tobramycin. Resistance to monobactams may also be explained by the production of isosulfazecin by strain ATCC 31433 (1). The biosynthetic gene cluster for this metabolite, like that for sulfazecin in P. acidophila (19), contains efflux pumps and a β-lactamase as self-resistance mechanisms.

Despite the potential pathogenicity suggested by the genome sequence, to our knowledge there are no reported cases of strain ATCC 31433 causing disease; however, opportunistic pathogenicity toward vulnerable hosts remains an inherent trait of the B. cepacia complex (9). Conversely, the large number of biosynthetic gene clusters identified in the genome of strain ATCC 31433 suggests that it may be a rich source of specialized metabolites, compared to related Burkholderia species, and B. ubonensis has been shown to have activity against the pathogen B. pseudomallei (45). AntiSMASH (16) analysis of the strain ATCC 31433 genome identified known gene clusters for malleilactone, ornibactin, isosulfazecin, alkylhydroxyquinoline, and pyrrolnitrin biosynthesis, while revealing additional biosynthetic potential from uncharacterized biosynthetic gene clusters for polyketides, nonribosomal peptides, and other metabolites. Genes encoding bacteriocins, which are thought to represent the active principle in B. ubonensis activity against B. pseudomallei (45), were found. The biosynthesis of bulgecin is of particular interest. The biosynthetic route to the bulgecinine (4-hydroxy-5-hydroxymethylproline) core of bulgecin (12, 46) is not known but may involve direct elaboration of proline or the action of a transketolase on hydroxypyruvate and aspartate semialdehyde, prior to transfer to NRPS, glycosyltransferase, and sulfotransferase components for formation of bulgecin A itself. Several candidate genes have been identified for further investigation.

In summary, the genome sequence of strain ATCC 31433 is consistent with that of an environmental Burkholderia isolate from the B. cepacia complex. The full biosynthetic potential of this strain, including determination of the location of the gene cluster responsible for biosynthesis of the bulgecins and identification of the products of the uncharacterized gene clusters, as well as precise classification of the strain, merits further investigation. Bulgecin may prove to be a valuable tool for studying and overcoming antibiotic resistance.

## MATERIALS AND METHODS

### Bacterial strains and susceptibility testing.

P. mesoacidophila ATCC 31433 was purchased from LGC Standards and was grown at 28°C in PS broth (1% [wt/vol] glucose, 0.5% [wt/vol] tryptone, 0.5% [wt/vol] meat extract, 0.5% [wt/vol] NaCl [pH 7.0]) except where indicated. Escherichia coli JM109 was purchased from Promega and was grown at 37°C in LB medium except where indicated. Antibiotics and metal salts were obtained from Sigma-Aldrich (Poole, United Kingdom) or Melford (Ipswich, United Kingdom). All antibiotic susceptibility testing followed the methods described by the BSAC (47), except that Mueller-Hinton II agar (Sigma-Aldrich) was used for disc diffusion assays and PS broth was used for broth microdilution assays. Breakpoints were taken from BSAC methods for antimicrobial susceptibility testing version 14 (30). For disc diffusion assays, antibiotic discs (a maximum of 3 per plate) were prepared using 5-mm filter paper discs impregnated with 5 μl of antibiotic stock solution. Metal susceptibility testing was performed only using broth microdilution assays, with 10, 1, or 0.001 mg ml^−1^ metal salt as the highest concentration on the plate. Antibiotic and metal susceptibility testing was performed at least in triplicate. Equivalent replicates for P. mesoacidophila and E. coli JM109 were performed on the same day using the same antibiotic stock solution.

### Genome analysis.

Genomic DNA was prepared from a 3-ml overnight culture of P. mesoacidophila in tryptic soy broth (Thermo Fisher Scientific). Cells were harvested by centrifugation and resuspended in 400 μl of 4 M guanidine isothiocyanate solution (Thermo Fisher Scientific). DNA extraction was performed using the Maxwell 16 automated nucleic acid purification system with the Maxwell Tissue DNA purification kit (Promega), following the manufacturer's instructions.

The genome sequence was assembled from data obtained from two SMRT cells, with a PacBio RSII sequencer, using Canu 1.3 (48), polished using Quiver 2.1.0 (Pacific Biosciences, Menlo Park, CA), and circularized, where possible, using Circlator 1.2.1 (49). Gene annotations were made using PROKKA 1.11 (50) and RAST (51–53). Biosynthetic gene clusters in the genome were analyzed using AntiSMASH 3.0 (16). Graphical genome comparisons were obtained with EasyFig 2.2.2, and the ANI was calculated with pyani 0.2.1. A virtual machine hosted by the Cloud Infrastructure for Microbial Bioinformatics (CLIMB) consortium (54) was used to perform genome assembly, annotation, and comparisons.

The sequences of six MLST alleles (*trpB*, *recA*, *lepA*, *gyrB*, *gltB*, and *atpD*) from P. mesoacidophila and six available B. ubonensis genomes were concatenated and aligned. Publicly available complete B. ubonensis genomes, deposited by Northern Arizona University, were downloaded from NCBI BioProject accession number PRJNA279182. The corresponding loci from 26 Burkholderia species were downloaded from the Burkholderia MLST database (http://pubMLST.org). A phylogeny was reconstructed with Geneious 7.1.9 Tree Builder, using the Tamura-Nei genetic distance model and the neighbor-joining tree-building method.

### Detection of inducible β-lactamase activity.

Starter cultures of P. mesoacidophila and E. coli JM109 were each subcultured (1:50 dilution) into four 100-ml portions of fresh PS broth and were grown at 28°C. Once *A*_600_ values of ∼0.4 were attained, cefoxitin was added to give final concentrations of 0, 1, 10, and 100 μg ml^−1^. Three hours after the addition of cefoxitin, a 25-ml aliquot was removed from each flask and the cells were lysed by sonication. The crude cellular lysate (100 μl) was then added to 200 μM nitrocefin solution in 50 mM sodium cacodylate buffer (pH 7.0) containing 100 μM ZnCl_2_ and 150 mM NaCl (900 μl), and the initial rate of hydrolysis was recorded by monitoring the decrease in absorbance at 482 nm. Three repeats were obtained for each initial rate of hydrolysis, and the entire experiment was performed in triplicate.

### Detection of bulgecin activity.

A 48-h culture of P. mesoacidophila in PS broth was subcultured (1:50 dilution) into PF broth (3% glycerol, 0.5% [wt/vol] tryptone, 0.5% [wt/vol] meat extract, 0.5% [wt/vol] NaCl, 0.1% [wt/vol] glucose, 0.1% [wt/vol] cysteine [pH 7.0]) and incubated for 78 h at 28°C, at 200 rpm. Cells were removed by centrifugation, and nutrients were replenished in the supernatant solution by the addition of 0.8% (wt/vol) nutrient broth 3 (i.e., 0.5% [wt/vol] tryptone and 0.3% [wt/vol] meat extract) (Sigma-Aldrich), 2% (wt/vol) glycerol, and 0.1% (wt/vol) glucose. All broths were readjusted to pH 7 and held at 4°C overnight.

An overnight culture of E. coli JM109 in LB medium was subcultured (1:50 dilution) into fresh LB medium and grown at 37°C to an *A*_600_ value of ∼0.3, at 250 rpm. Cultures were centrifuged and the cell pellets were resuspended in the test broths. Cultures were then grown for an additional 4 h at 37°C, at 250 rpm. Cefuroxime (final concentration, 5 μg ml^−1^) was added to one of each pair of flasks after 30 min; 60 and 90 min after cefuroxime addition, aliquots were removed and cells were viewed by light microscopy using an Ultraphot microscope (Zeiss). These experiments were repeated four times.

### Accession number(s).

The assembled genome sequence has been deposited at GenBank under accession numbers CP020737 to CP020740. The corresponding raw sequence read data are available from the European Nucleotide Archive under accession number ERP022292.
